# Trends and hotspots in the field of diabetic retinopathy imaging research from 2000–2023

**DOI:** 10.3389/fmed.2024.1481088

**Published:** 2024-10-09

**Authors:** Qing Zhang, Ping Zhang, Naimei Chen, Zhentao Zhu, Wangting Li, Qiang Wang

**Affiliations:** ^1^The Third Affiliated Hospital of Xinxiang Medical University, Xinxiang Medical University, Xinxiang, China; ^2^Shenzhen Eye Institute, Shenzhen Eye Hospital, Jinan University, Shenzhen, China; ^3^Department of Ophthalmology, Huaian Hospital of Huaian City, Huaian, China; ^4^Department of Ophthalmology, Third Affiliated Hospital, Wenzhou Medical University, Zhejiang, China

**Keywords:** diabetic retinopathy, imaging, diagnosis, artificial intelligence, bibliometric

## Abstract

**Background:**

Diabetic retinopathy (DR) poses a major threat to diabetic patients’ vision and is a critical public health issue. Imaging applications for DR have grown since the 21st century, aiding diagnosis, grading, and screening. This study uses bibliometric analysis to assess the field’s advancements and key areas of interest.

**Methods:**

This study performed a bibliometric analysis of DR imaging articles collected from the Web of Science Core Collection database between January 1st, 2000, and December 31st, 2023. The literature information was then analyzed through CiteSpace.

**Results:**

The United States and China led in the number of publications, with 719 and 609, respectively. The University of London topped the institution list with 139 papers. Tien Yin Wong was the most prolific researcher. Invest. Ophthalmol. Vis. Sci. published the most articles (105). Notable burst keywords were “deep learning,” “artificial intelligence,” et al.

**Conclusion:**

The United States is at the forefront of DR research, with the University of London as the top institution and Invest. Ophthalmol. Vis. Sci. as the most published journal. Tien Yin Wong is the most influential researcher. Hotspots like “deep learning,” and “artificial intelligence,” have seen a significant rise, indicating artificial intelligence’s growing role in DR imaging.

## 1 Introduction

Diabetic retinopathy (DR), as a microvascular complication of diabetes, poses a serious threat to patients’ vision health. With the sharp increase in the number of diabetic patients worldwide, the prevention, diagnosis, and treatment of DR has become a focus of concern in the medical community and the public health field (1). Since the 21st century, the rapid development of medical imaging technology has greatly promoted the clinical diagnosis and management of DR. Particularly, Optical Coherence Tomography (OCT) (2), Fluorescein Angiography (FA) (3), and Optical Coherence Tomography Angiography (OCTA) have become indispensable tools for the diagnosis of DR (4). Moreover, with the development of artificial intelligence (AI) technologies such as deep learning (DL), the application prospects of AI technology in the field of DR imaging are broad, not only improving the efficiency and accuracy of diagnosis but also helping to achieve personalized management, optimize clinical decision-making, and promote the progress of prevention and management of diabetic retinopathy (5).

A large number of bibliometric studies related to DR have been conducted in the past, providing new ideas and insights for exploring the field of ophthalmology (6). DR imaging research is an important part of DR research, and in recent years, there has been a continuous emergence of research results related to DR imaging. It is necessary to conduct systematic and comprehensive bibliometric research. This study aims to conduct the latest bibliometric research on the literature information of conventional imaging research directions of DR while combining the most influential papers in this field to assess and analyze the hotspots and trends of DR imaging.

## 2 Materials and methods

Bibliometric analysis was conducted by determining the search strategy and selecting publications. The search strategy included limiting databases, search terms, languages, document types, and publication dates. This paper selected the Web of Science Core Collection (WoSCC) as the source for bibliometric data, which covers a variety of research journals and provides various bibliometric indicators (such as titles, institutions, countries/regions, publication years, categories, and keywords). To ensure the accuracy and authority of the retrieved data, the indices selected were SCI-Expanded and SSCI.

Considering the history of DR imaging research, the search strategy for this study was TS = ((“diabetic retinopathy”) AND (“imag*” OR “phot*” OR “OCT” OR “Optical Coherence Tomography” OR “Optical Coherence Tomography Angiography” OR “fluorescence angiography” OR “FFA”)) AND “publication year”: 2000–2023 AND “Document type”: Article AND “Language”: English. The document type was selected as article, and the language type was English.

**Figure 1:**
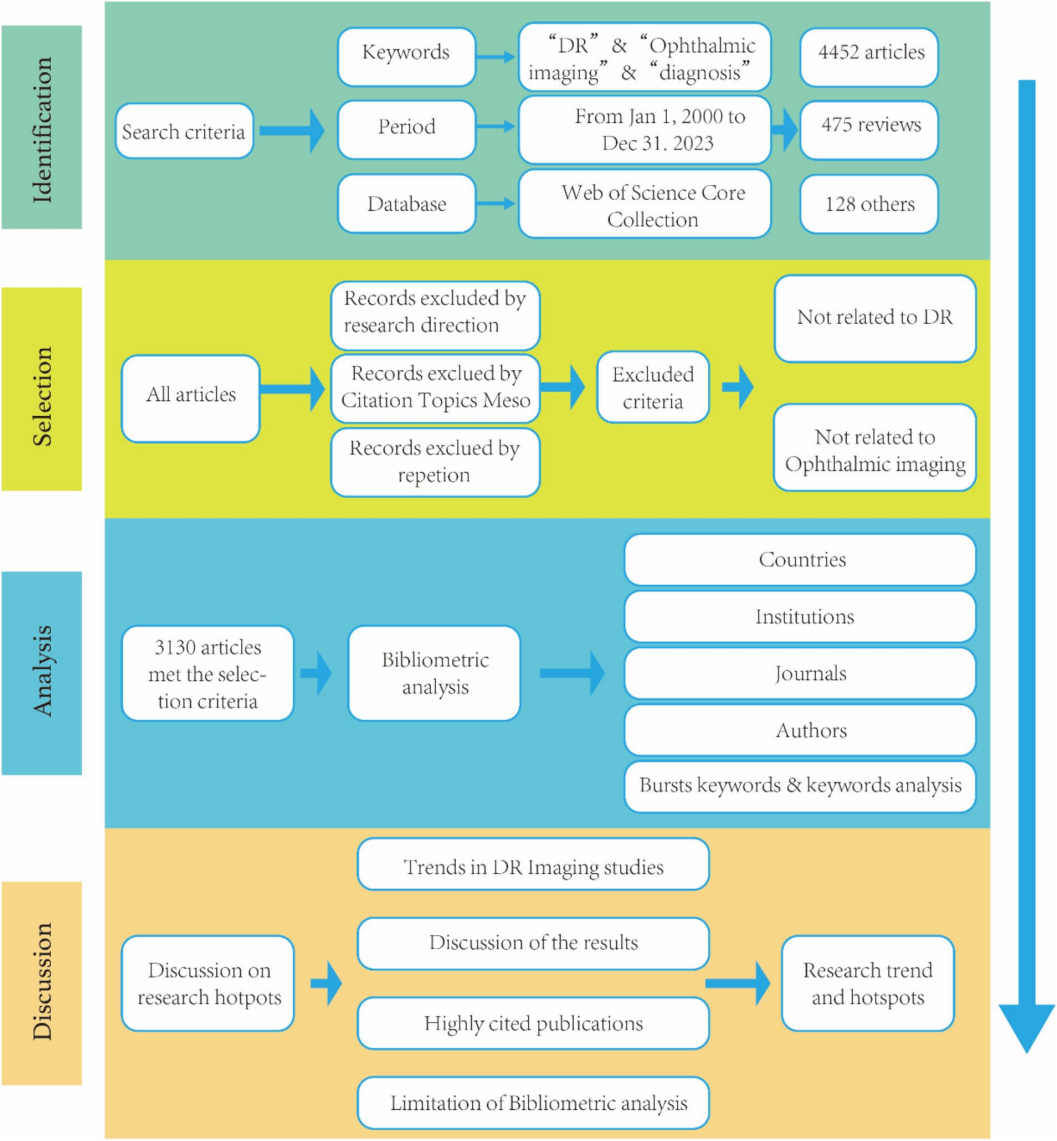
Frame flow diagram.

During the data screening process, two professional ophthalmic researchers checked the data separately. Studies unrelated to DR imaging were excluded from the search process. Subsequently, bibliometric and visualization analyses were conducted on the included publications. The detailed search and analysis process is shown in Figure 1. In the data analysis, CiteSpace 6.2.R4 and the functions of the WoS website were used to analyze the collaboration networks and H-indexes of countries or regions, institutions, authors, journals, keywords, and research categories. The H-index serves as a quantitative indicator to assess a researcher’s or research institution’s academic influence and productivity by reflecting the volume and citation impact of their published work.

## 3 Results

### 3.1 Distribution of articles by year

**Figure 2:**
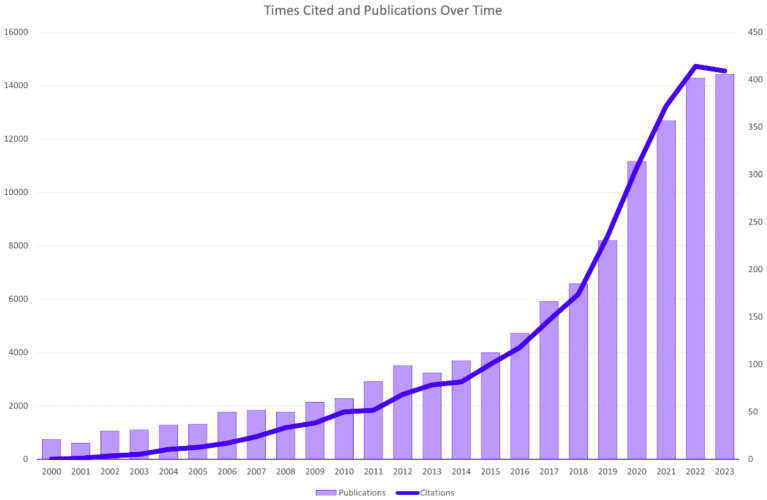
Times cited and publications over time.

After the above identification and screening, it was determined that this study included 3,130 publications, which have been cited 104,234 times over the past 24 years, with an average of 33.3 citations per publication. Figure 2 shows the temporal distribution of publication volume and worldwide citation volume in domestic and international ophthalmic AI research. The overall upward trend of papers published in this field has been maintained since the beginning of the 21st century. Globally, the publication volume has exceeded 400 papers in each of the past 2 years. The citation volume of research papers in this field has shown an overall increasing trend year by year.

### 3.2 Countries or regions

**Figure 3:**
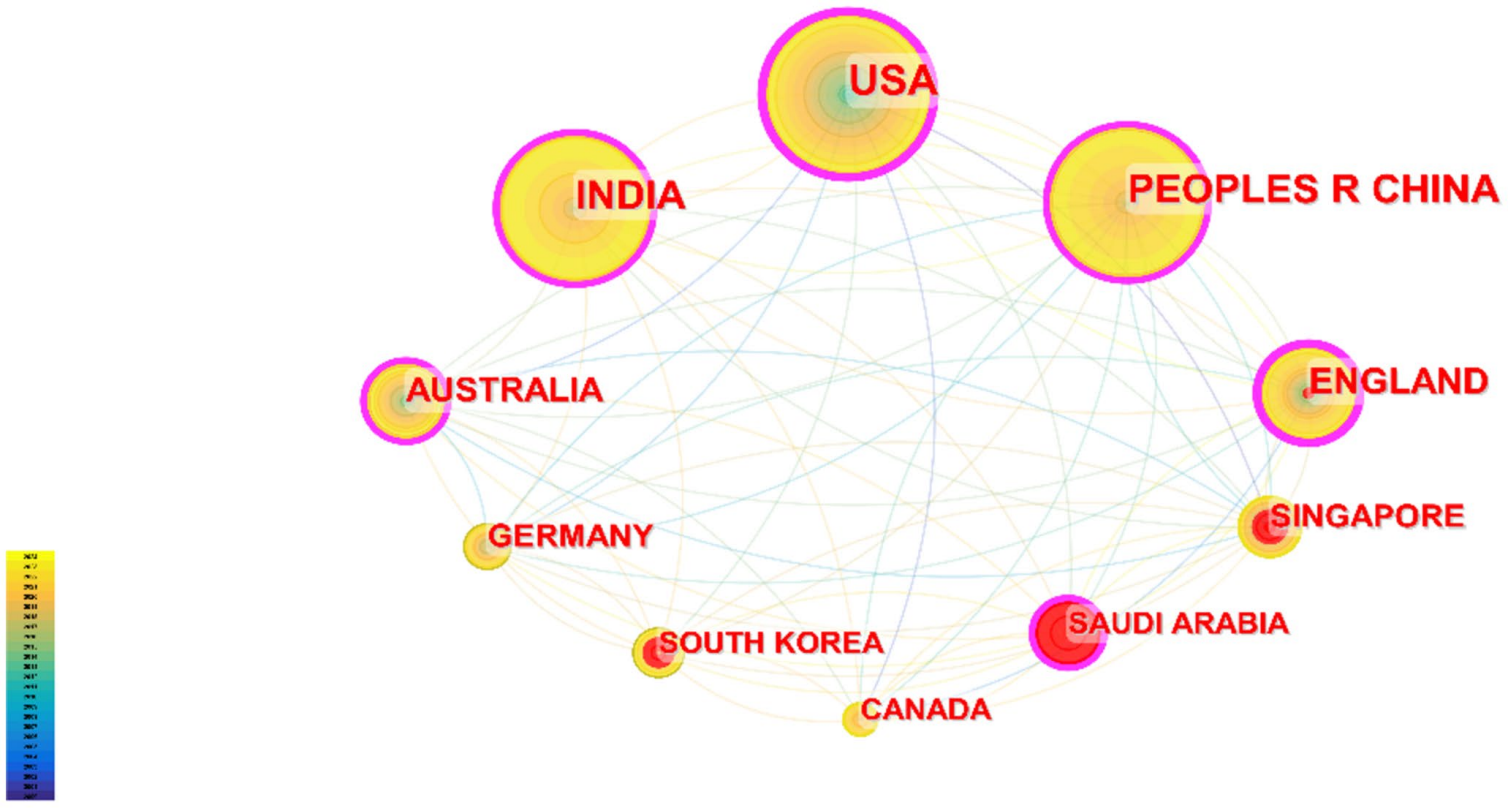
Cooperation of countries or regions.

These publications come from 105 countries and regions. Figure 3 shows the collaboration network map of each country or region, where the size of each label and node area is proportional to the number of publications. The United States (719), China (609), and India (514) have the largest label and node areas, indicating the highest volume of publications. The connections between nodes imply collaboration between countries or regions; more connections indicate closer cooperation. Table 1 provides details of the top 10 countries or regions in terms of the number of papers published, including Centrality reflecting their collaboration strength and the H-index measuring their influence (7). The H-indexes of the United States and China are 97 and 60, respectively.

**Table 1:** Top 10 countries or regions with articles on DR imaging.

Rank	Country/Region	Counts	Centrality	H-index
1	United States	719	0.24	97
2	China	609	0.17	60
3	India	514	0.19	50
4	England	308	0.31	56
5	Australia	217	0.17	54
6	Singapore	152	0.04	52
7	Germany	132	0.07	36
8	Saudi Arabia	107	0.16	24
9	Korea	105	0.02	31
10	Canada	95	0.06	32

### 3.3 Institutions and researchers

Table 2 ranks the top 10 global institutions by the number of papers published, with the publication volume of the top 10 institutions all exceeding 90 papers, and the H-index of the top 6 institutions all exceeding 30. Similarly, we also ranked the institutions with the highest number of publications in China, see Table 3. The top three institutions in terms of publication volume are the University of London (139), University College London (117), and the National University of Singapore (112), with the H-index of the top 10 institutions all exceeding 30.

**Table 2:** Top 10 Institutions with articles on DR imaging.

Rank	Affiliation	Counts	H-index
1	University of London	139	39
2	University College London	117	39
3	National University of Singapore	112	48
4	Moorfields Eye Hospital NHS Foundation Trust	102	36
5	Singapore National Eye Center	102	46
6	Harvard University	95	34
7	University of Wisconsin System	89	41
8	Harvard Medical School	86	32
9	University of Melbourne	84	47
10	University of California System	83	32

**Table 3:** Top 10 Researchers with articles on DR imaging.

Rank	Researcher	Counts	H-index
1	Tien Yin Wong	91	48
2	Klein, Ronald	59	37
3	Rajiv Raman	47	19
4	Jie Jin Wang	44	31
5	Tunde Peto	43	19
6	S. Sivaprasad	41	15
7	Aiello, Lloyd Paul	35	23
8	Paolo Silva	33	16
9	Paul Mitchell	29	21
10	Cunha-Vaz, Jose	28	13

Among domestic and international researchers, Tien Yin Wong, Klein, Ronald, and Rajiv Raman all have more than 45 publications, with Tien Yin Wong, Klein, Ronald, and Jie Jin Wang having high H-indexes of 48, 37, and 31, respectively. Table 4 ranks global researchers by the number of papers published.

**Table 4:** Top 10 Journals with articles on DR imaging.

Rank	Journal	Counts	Category	H-index	IF (2023)
1	Invest. Ophthalmol. Vis. Sci.	105	Ophthalmology	47	5
2	Retin.-J. Retin. Vitr. Dis.	98	Ophthalmology	33	2.3
3	Br. J. Ophthalmol.	97	Ophthalmology	33	3.7
4	Ophthalmology	94	Ophthalmology	55	13.1
5	IEEE Access	77	Computer Science/Engineering/Telecommunications	27	3.4
6	PLoS One	73	Multidisciplinary Sciences	28	2.9
7	Eye	70	Ophthalmology	22	2.8
8	Multimed. Tools Appl.	61	Computer Science/Engineering	13	3
9	Transl. Vis. Sci. Technol.	61	Ophthalmology	17	2.6
10	Am. J. Ophthalmol.	60	Ophthalmology	32	4.1

### 3.4 Journals and categories

The 3,130 papers included in this study were published in 649 journals. Table 4 lists the top 10 journals by publication volume and summarizes the research directions, H-indexes, and impact factors (IFs) for 2023 of the 10 major academic journals. The two journals with the highest publication volumes are Invest. Ophthalmol. Vis. Sci. with 105 publications and Retin.-J. Retin. Vitr. Dis. with 98; their H-indexes are 47 and 33 respectively, and their IFs are 5.0 and 2.3, respectively. Since this field is related to ophthalmology and imaging research, most of the top 10 journals by publication volume are ophthalmology journals, with additional journals covering fields such as Computer Science/Engineering.

### 3.5 Keywords

**Figure 4:**
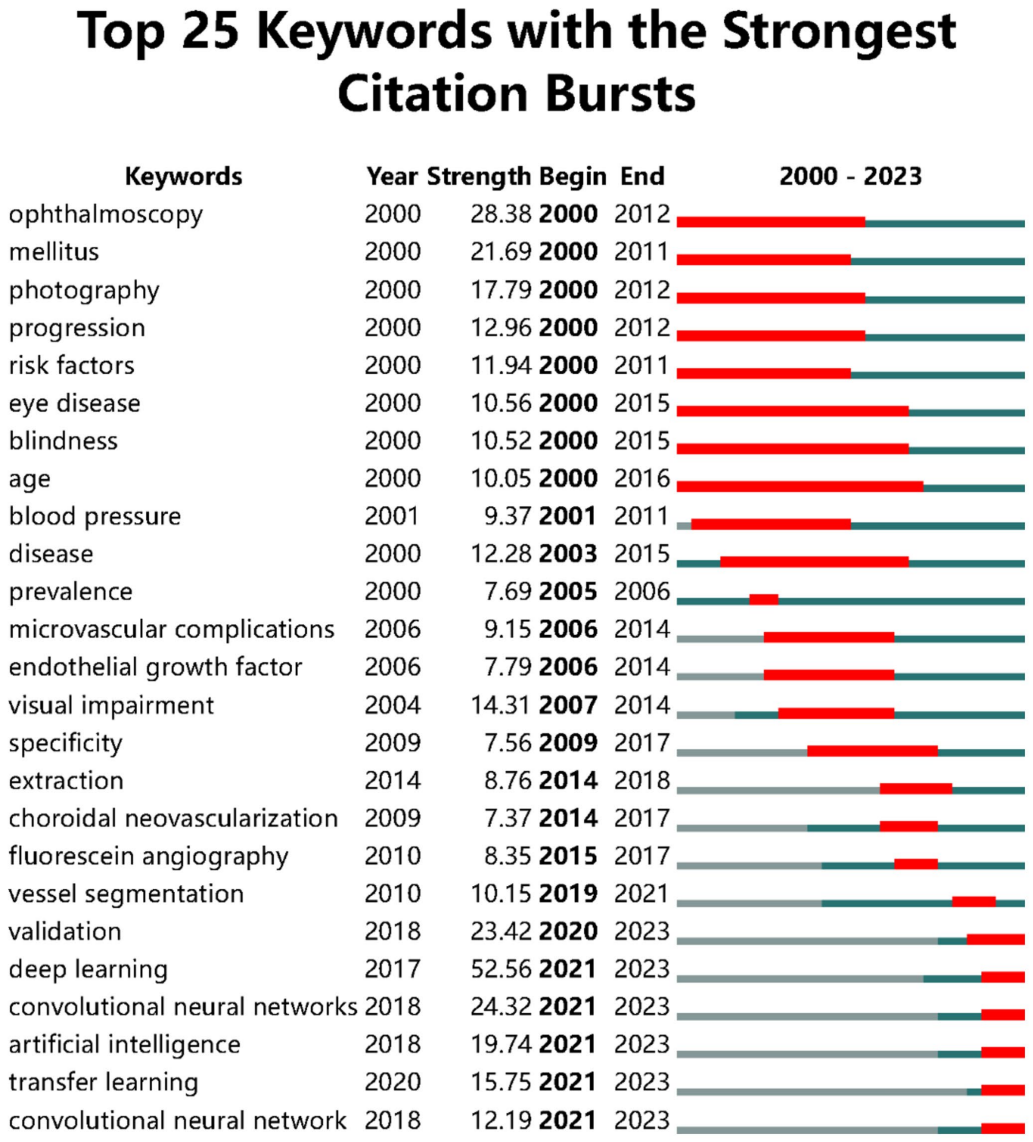
Keywords with the strongest citation bursts.

Using CiteSpace for co-citation analysis of keywords, we selected the keywords of the literature information for text processing and analysis with parameters set as Timespan: 2000–2023 (Slice Length = 1), Selection Criteria: g-index (k = 25). Figure 4 highlights the top 25 most cited keywords, whose intensity indicates the time and strength of the appearance of the keywords, with red squares representing the timeline of the keyword surge. “Age” is the keyword with the longest active time, and the keywords that have surged in the past 3 years are “deep learning,” “convolutional neural networks,” “artificial intelligence,” and “transfer learning.” The keyword with the strongest surge intensity is “deep learning”.

### 3.6 High-impact articles

Highly cited article signifies that the research has a high impact and also indicates widely recognized research achievements in the field. At present, the research contents of highly cited documents are mainly distributed in AI research, clinical trial, cross-sectional study, and so on. Table 5 lists the 10 most cited articles in the field of diabetic retinopathy imaging.

**Table 5:** High-impact articles.

Rank	Title	Cited times	Research Content	Limitations
1	Development and Validation of a Deep Learning Algorithm for Detection of Diabetic Retinopathy in Retinal Fundus Photographs. Gulshan et al. (9)	3,732	In this study, researchers have developed a Deep Learning algorithm system, which has high sensitivity and specificity in detecting DR.	1. The reference criteria are based on the majority decision of ophthalmologist graders this means that the algorithm may not be able to handle images with subtle findings very well; 2. The algorithm only provides the grade of the image and does not directly define the fundus image; 3. This algorithm is not a substitute for a complete eye examination; 4. The algorithm is using features formerly unrecognized and ignored by humans.
2	Ridge-based vessel segmentation in color images of the retina. Staal et al. (8)	2,528	Researchers have proposed a method for automatic segmentation of blood vessels in retinal color fundus images using supervised methods, which has certain value in screening DR.	1. Labeling data is laborious and time-consuming; 2. This research requires large amounts of training data to increase its “learning” power, so it is important to balance computational resources with method performance; 3. Because of human subjectivity, it is difficult to grasp the accuracy mark of the edge blood vessels, which affects the processing of marginal blood vessels by segmentation methods.
3	Development and Validation of a Deep Learning System for Diabetic Retinopathy and Related Eye Diseases Using Retinal Images From Multiethnic Populations With Diabetes. Ting et al. (70)	1,206	In this study, the development and validation of DLS by using retinal images of multi-ethnic diabetic patients, which showed high sensitivity and specificity in identifying DR and eye-related diseases.	1. Reference standards for external datasets are based on the different assessments of people from various ophthalmological backgrounds; 2. The multilevel analyses of DLS are not consistent with the clinical diagnosis of disease signs, and these issues may have an impact on physicians’ acceptance for clinical use; 3. DLS still relies on OCT technology and other ophthalmic examinations to identify fundus cases.
4	Prevalence of Diabetic Retinopathy in the United States, 2005–2008. Zhang et al. (27)	800	A cross-sectional study of US adults aged 40 years and older with Diabetes, aiming to obtain an analysis of the prevalence, severity, and related risk factors of Diabetic Retinopathy among diabetics from 2005 to 2008.	1. The study could not distinguish between type 1 and type 2 diabetes and assess the specific risk of other retinal diseases in diabetic patients; 2. Some patients were excluded from the study due to lack of data, which may bias estimates of diabetic retinopathy prevalence; 3. Other racial subgroups were not described in this study; 4. Due to the small sample size, we may not be able to detect differences between and among subgroups.
5	Effect of fenofibrate on the need for laser treatment for diabetic retinopathy (FIELD study): a randomized controlled trial. Keech et al. (75)	777	This study describes a clinical trial called the FIELD study, to Explore the impact of long-term lipid-lowering therapy with fenofibrate on disease progression and the need for laser therapy in DR patients.	1. In the study about 10% of patients receiving laser treatment, their data were collected retrospectively, however, follow-up data loss is large; 2. Only fundus pictures were taken in a small sample of patients, affecting the judgment of lesion progression before and after laser treatment.
6	Feedback on a publicly distributed image database the MESSIDOR database. Decenciere et al. (76)	739	Six years after the MESSIDOR database was made public, the researchers conducted statistical analyses of the database’s usage and user feedback.	Based on the existing experience, the researchers provide valid advice for the design of future databases but do not provide concrete and feasible recommendations for the updating, management and operation of this database.
7	Intravitreal triamcinolone for refractory diabetic macular edema. Martidis et al. (77)	711	Exploring the improvement of macular edema in patients with after intravitreal injection of triamcinolone for laser photocoagulation non-responsiveness DME.	1. The sample size of this study is small and its efficacy and safety need to be evaluated for the future; 2. Visual acuity was not measured on the more accepted ETDRS chart; 3. This study had a short follow-up period; 4.in this study, a control group was not clearly established.
8	Automated Identification of Diabetic Retinopathy Using Deep Learning. Gargeya et al. (71)	709	This study introduces a competitive Deep Learning grading system, which can be used to achieve the purpose of DR Screening and timely transfer of patients.	1. The automated diagnostic system relies on high-definition retinal fundus images; 2. The algorithm is just an auxiliary diagnostic tool, the final diagnosis of the disease still needs professional ophthalmologist’s evaluation; 3. The output of this study is binary and does not reflect the severity of the disease.
9	UKPDS 50: Risk factors for incidence and progression of retinopathy in Type II diabetes over 6 years from diagnosis. Stratton et al. (78)	695	Clinical analysis of newly diagnosed Type II diabetic patients in the UK by using prospective longitudinal data, with the aim of exploring risk factors for progression and DR development after 6 years of follow-up.	/
10	A New Supervised Method for Blood Vessel Segmentation in Retinal Images by Using Gray-Level and Moment Invariants-Based Features. Marín et al. (72)	658	This study presents a new supervised method for vascular detection on fundus images, based on neural networks. The method has been evaluated on open databases, demonstrating high accuracy.	1. The performance and safety of this method need to be further evaluated for practical clinical application; 2. When trained and tested on different databases, the performance results have degraded.

## 4 Discussion

### 4.1 Overall data

In the development of DR imaging and its clinical application in ophthalmology, we have observed an increase in publications in the field of AI in ophthalmology since the 21st century, with continuous explosive growth in recent years, as shown in Figure 2. As early as 2004, a high-impact paper on a retinal image diagnostic system for screening DR was published (8), and in 2016, numerous studies on the application of AI in the diagnosis and screening of ophthalmic diseases began to be published, with the most representative being Webster, R et al.’s use of DL to create an automatic detection algorithm for diabetic retinopathy and diabetic macular edema in retinal fundus photographs (9). Starting in 2017, research on the application of AI in DR imaging worldwide began to show a sustained upward trend. Nowadays, AI has developed a series of models for diagnosing and managing DR by processing and analyzing various modalities of data, such as fundus color photographs, OCT, OCTA, ocular ultrasound, and ocular parameters (10, 11). Nowadays, multimodal imaging data plays an increasingly important role in the diagnosis and grading of diabetic retinopathy (12). At the same time, multimodal imaging data can be used to train AI algorithms to automatically diagnose and grade DR, improving diagnostic efficiency and accuracy (13).

Looking at the global distribution of publication areas, the United States leads with 719 publications, and five countries in the Asian region are on the list, which may be related to the high population density and incidence of eye diseases in Asia (14). The United States, China, and India are leading in the field of DR imaging research, while England, although not the highest in publication volume, has a very high impact and quality of research. Other countries such as Australia, Singapore, Germany, etc., also have significant contributions in this field, but compared with the top countries, there is still room for improvement in their research impact and academic accumulation.

In the analysis of research institutions, we found that the top 10 institutions in terms of publication volume all have an H-index greater than 30. The University of London ranks first with 139 publications and an H-index of 39, indicating that the institution has a high level of academic output and research quality in the field of DR imaging research. From the results of bibliometric analysis, the University of London and University College London have closely cooperated in most studies. The University of Melbourne has the highest H-index, indicating that the university has a very high research quality and influence in this field. Harvard University and its medical school, the National University of Singapore, and the Singapore National Eye Center also demonstrate their strong research capabilities in this field. In addition, institutions such as the University of Wisconsin System and the University of California System also have significant contributions in this field.

Wong TY and Klein R have a significant leadership position in the field of DR imaging research. Their research is not only numerous but also of high quality and has had a profound impact on the academic community. Other researchers such as Raman R, Wang JJ, have also made important contributions in this field, but compared with the former two, their H-index is lower, which may mean that their research impact in the academic community still has room for improvement. By analyzing the publication output of the top three researchers, we can determine that Tien Yin Wong’s research focuses on the epidemiology, clinical treatment, and disease mechanisms of diabetic retinopathy (15–17). He has made significant achievements in the field of artificial intelligence research for diabetic retinopathy, developing advanced image analysis algorithms for automated detection and grading of retinal lesions, which help to improve the accuracy and efficiency of diagnosis (18). Klein, Ronald also has a high output in this field and has collaborated with Tien Yin Wong on numerous studies related to the imaging of diabetic retinopathy (15, 19, 20). Rajiv Raman has extensive experience in ophthalmic imaging and artificial intelligence research for diabetic retinopathy. His research team has developed various algorithms and software to improve the screening and management of diabetic retinopathy. These technologies identify signs of lesions by analyzing retinal images, providing possibilities for early intervention (21, 22). The work of these three top researchers indicates that artificial intelligence and machine learning technologies are playing an increasingly important role in the diagnosis and management of ophthalmic diseases. Their research not only improves the accuracy of disease diagnosis but also offers possibilities for the development of new treatment methods. The above information is very useful for assessing the academic status and influence of researchers and institutions, and can guide future research cooperation and academic exchanges.

From Table 5, it can be observed that journals in the field of ophthalmology hold a dominant position in DR imaging research, with the journal “Ophthalmology” demonstrating its leading status in the field due to its high H-index and impact factor. Concurrently, journals in the field of computer science, such as “IEEE Access” and “Multimedia Tools and Applications,” also play a role in DR imaging research, likely related to the reliance on image processing and analysis techniques in DR imaging research.

In the clustering of research fields, we find that this field of study encompasses Ophthalmology, Endocrinology and Metabolism, Computer Science, Information Systems, Health Care Sciences and Services, etc. Journals in the ophthalmology field, such as “Investigative Ophthalmology and Visual Science” and “Ophthalmology,” have a high volume of publications in DR imaging research, with high H-index and impact factor, reflecting the depth and breadth of research in this domain. The field of endocrinology and metabolism focuses on the management and treatment of diabetes, utilizing ophthalmic imaging tools to prevent and control the progression of DR. With technological advancements, computer science plays an increasingly vital role in DR imaging research. Technologies such as computer vision, machine learning, and AI are employed to enhance the automatic detection and analysis capabilities of DR. The field of health sciences and services is concerned with providing and improving medical services, including DR screening, patient care, health policy, and the organization of healthcare systems.

Ophthalmic research primarily focuses on the diagnosis, treatment, and preventive strategies of diseases, including the application of imaging technology in diagnosing DR. With the aid of AI, technologies like DL, image processing, and automatic segmentation are transforming the diagnosis and management paradigms of DR. Additionally, telemedicine (23), new types of ophthalmic imaging detection instruments, and interdisciplinary collaboration in computer science (24), the integration of interdisciplinary cooperation and technology has broadened the scope and advanced the development of DR imaging research.

### 4.2 Hotspots of DR imaging research

The distribution of hotspots in DR imaging research has continuously evolved over time. Initially, diagnoses and grading of DR were predominantly conducted through ophthalmoscopy (25). In the early stages of research on the automatic diagnosis of DR, models for the automatic diagnosis and grading of DR were validated using ophthalmoscopy results (26). Ophthalmoscopy, as an essential method in the clinical diagnosis and grading process of DR, has always played a crucial role in this field. However, the decline in its surge intensity over time may be related to the emergence of new diagnostic technologies. “Mellitus,” as the primary cause of DR, maintained a high level of keyword intensity between 2000 and 2011. During a similar period, research related to “Risk factors” and “Progression” received widespread attention. “Age” and “Blood Pressure” as significant risk factors for DR have been the focus of many researchers (27). The activity of “Visual impairment” and “Blindness” reflects the impact of DR as a leading cause of blindness, possibly indicating an increased focus on research into diabetes itself and its complications at the time. “Photography” maintained a high level from 2000 to 2012 and then declined, likely related to the rise of more advanced imaging technologies such as OCT and OCTA. The emergence and development of OCT and OCTA technologies have significantly propelled the progress of DR imaging, offering new perspectives and tools for the diagnosis, treatment, and research of DR (28).

OCT has revolutionized the diagnosis and research of DR with several key advancements: 1. Non-invasive imaging: OCT’s non-invasive approach, eliminating the need for fluorescent dyes, facilitates high-resolution retinal imaging. This technology is crucial for observing microvascular changes in DR, such as microaneurysms, macular edema, and neovascularization (29). 2. OCTA: As a derivative of OCT technology, OCTA offers a non-invasive method to image retinal vasculature with superior contrast and resolution compared to traditional fluorescein angiography (4). It enables quantitative analysis of capillary changes, serving as an objective diagnostic and therapeutic assessment tool for DR (30). 3. Wide-field imaging: Advances in OCTA have expanded the imaging field, allowing for a comprehensive assessment of retinal abnormalities in DR, even those beyond the macular region. This enhancement is vital for treatment monitoring, particularly in evaluating responses to anti-VEGF and laser therapies, by detailing retinal structural and vascular modifications. 4. Early diagnosis and lesion monitoring: OCTA’s ability to detect early microvascular alterations facilitates timely diagnosis and ongoing lesion surveillance, essential for proactive intervention strategies (31).

Wide-field imaging and Adaptive Optics (AO) imaging are integral to DR diagnosis and research, yet they face specific limitations and challenges. Wide-field imaging extends the retinal view beyond the capabilities of conventional fundus cameras, aiding in the detection of peripheral retinal lesions in DR (32). Despite its broader view, it suffers from reduced resolution in peripheral details and potential image distortion, which can impair accurate lesion assessment. Future improvements may focus on enhancing image quality and resolution, as well as refining image processing algorithms to minimize distortion (33). AO imaging, initially developed for astronomy, has been adapted for ophthalmology to correct ocular optical aberrations, offering high-resolution retinal imaging down to the photoreceptor level. Despite its potential, AO imaging is hindered by technical complexity, high costs, and limited clinical adoption. Future developments should aim to simplify system design, reduce costs, and improve imaging speed and stability to increase its clinical utility (34, 35).

In recent years, with the development of AI applications in ophthalmology, neural network models have played a crucial role. They mainly use DL algorithms such as convolutional neural networks (CNN) to analyze retinal images and other ocular examination images for the diagnosis, classification, and prediction of retinal diseases. Neural network models learn from massive amounts of data, enabling the identification of complex patterns and features, allowing physicians to more accurately assess patients’ retinal images (36). DL algorithms can identify characteristics of DR, such as microaneurysms, hemorrhages, and exudates, by analyzing a large number of fundus image data, with accuracy in some cases comparable to that of professional ophthalmologists (37). DL aids in the early automatic screening of DR, which is crucial for preventing vision loss, especially in areas lacking professional medical resources (38). In DR imaging research, “validation” is a key step to ensure the accuracy, reliability, and generalization capability of the research model. When developing imaging models for DR detection, researchers need to ensure that the model can accurately identify and classify different stages of retinal lesions through the validation process. This usually involves using an independent test set that does not participate in the model’s training process (39); to improve the reliability of model validation, researchers may use cross-validation techniques, such as k-fold cross-validation, which helps to evaluate the model’s performance on different data subsets and reduces the risk of overfitting (40); validation of DR imaging research models may also involve multicenter data to ensure that the model maintains consistent performance under different medical environments and equipment (41). In addition to technical validation, the model also needs to be validated in a clinical setting to ensure its effectiveness and feasibility in practical applications. This may include comparing the model’s diagnostic results with those of clinical physicians and assessing the model’s impact on patient management (40). In actual clinical work, real-time validation of continuous data flow is also needed to evaluate the model’s generalization ability to different populations (such as different ages, genders, races), ensuring the model’s wide applicability (9). As a special method and extension of “machine learning (ML),” “deep learning” and “transfer learning” have continued to surge in recent years, reflecting the importance of ML in the field of AI for DR imaging. By automatically recognizing and quantitatively assessing lesions, ML can accurately and quickly diagnose ophthalmic diseases such as diabetic retinopathy (42), age-related macular degeneration (43), and glaucoma (44). In addition, ML is playing an increasingly growing role in the establishment of personalized treatment plans in ophthalmology, monitoring disease progression, and evaluating therapeutic effects (45).

The field of DR imaging research is gradually shifting from traditional diagnostic methods to utilizing advanced computer vision and AI technologies to improve the accuracy and efficiency of diagnosis. The application of these technologies not only helps to better understand the pathophysiological mechanisms of DR but may also provide new tools for early diagnosis and personalized treatment.

### 4.3 Trends of DR imaging research

With the advent of the artificial intelligence era, various AI researches related to DR imaging have emerged, focusing on the development of multi-classification models for DR, multi-disease models, AI-assisted prediction and management of DR progression, the application of clinically qualified AI models, and real-world studies of AI models in practice.

In the realm of DR imaging diagnosis, multi-classification AI models excel by providing detailed disease grading, enhancing diagnostic precision, automating large-scale image processing, and exhibiting robust generalization. Recent studies have extensively explored DR classification using pretrained models like ResNet50 and VGG-16, employing techniques such as image preprocessing, data augmentation, and model fine-tuning to boost classification accuracy (46–48). VGG-16 has been particularly effective in managing imbalanced datasets and achieving high accuracy. In contrast, ResNet50 has shown sensitivity to input resolution, loss functions, and data augmentation strategies, with mean square error optimization enhancing specific evaluation metrics (49, 50). Moreover, the incorporation of deep learning techniques such as batch normalization, dropout layers, and learn-rate scheduling has significantly improved the classification accuracy of DR severity (48). These advancements underscore the pivotal role of AI in accurately classifying DR, facilitating its early detection and intervention.

Multi-disease diagnostic models offer a paradigm shift by enabling concurrent analysis of multiple conditions, thereby streamlining the diagnostic process and reducing the need for repetitive medical examinations. The CARE system, a pioneering real-world study project for AI in ophthalmology, can identify a spectrum of 14 common fundus diseases through fundus photographs, achieving an impressive diagnostic accuracy rate of 0.952 (51). VisionFM, trained on a vast dataset of 3.4 million images from over half a million individuals, covers a diverse range of ophthalmic conditions and imaging modalities. It serves as a cornerstone for applications in disease screening, diagnosis, prognosis, and phenotype subdivision, demonstrating exceptional performance in the concurrent diagnosis of 12 prevalent ophthalmic diseases (52). Furthermore, researchers are delving into the potential of ophthalmic imaging for diagnosing systemic diseases beyond the eye (53). The evolution and deployment of these AI models highlight their transformative impact on diagnostic efficiency, cost reduction, and the equitable distribution of medical resources in ophthalmology and beyond.

AI’s ability to predict future outcomes by recognizing patterns in past data is often more efficient than that of experienced human experts. Studies have shown that AI can predict the development of referable DR from fundus imagery with high accuracy at both the individual image and patient levels (54, 55). Additionally, AI models based on quantitative OCT imaging biomarkers have been successful in predicting visual outcomes and treatment needs in neovascular age-related macular degeneration (nAMD) patients, supporting the management of active and progressive nAMD with AI assistance (56). These predictive capabilities of AI in treatment and prognosis can offer significant insights for optimizing patient care, potentially leading to cost savings in healthcare.

The transition of AI models from research to the clinical marketplace has been marked by significant advancements in DR imaging diagnosis. Notable systems such as IDx-DR by Digital Diagnostics and EyeArt by Eyenuk Inc. have received approval from the U.S. Food and Drug Administration (FDA). IDx-DR, the first AI-driven DR diagnostic product approved in 2018, assesses DR severity from retinal photographs with high sensitivity and specificity nearing 90%. EyeArt stands out for its ability to analyze data from 100,000 individuals within 45h, highlighting the transformative impact of AI on clinical efficiency in DR diagnosis (57, 58).

Real-world studies on AI for DR screening have shown promising results. These studies have demonstrated high diagnostic accuracy in detecting referable DR, with pooled sensitivity ranging from 91.24 to 95.33% and specificity from 92.01 to 93.90% (59). AI algorithms, like Selena+, have been validated in clinical settings, showing sensitivity and specificity of 96.8% for DR detection (60). AI-based systems have also been developed to assist in DR grading, achieving high accuracy values and improving the diagnostic performance of medical students and junior residents (61). Furthermore, AI-assisted DR screening models have been implemented in Australian healthcare settings, demonstrating an area under the curve of 0.92, sensitivity of 96.9%, and specificity of 87.7%, with high patient and clinician satisfaction rates (62). However, challenges remain in achieving higher specificity to reduce unnecessary referrals and burden on healthcare providers (63).

Through numerous studies, AI models analyze retinal and other ophthalmic examination images, facilitating the diagnosis, classification, and prediction of retinal diseases. By learning from vast datasets, these models identify complex patterns and features, thereby enabling physicians to assess retinal images more accurately. In studies, DL algorithms have been shown to identify DR features such as microaneurysms, hemorrhages, and exudates with high accuracy, comparable to professional ophthalmologists. Moreover, AI plays a crucial role in the early automatic screening of DR, which is essential for preventing vision loss, especially in regions with limited medical resources. The development and real-world application of clinically qualified AI models are gaining traction, highlighting AI’s potential to enhance the accessibility and efficiency of DR screening (64).

In the context of multimodal DR models, AI is utilized to analyze diverse data patterns, including color fundus photographs, OCT, OCTA, ocular ultrasound, ocular parameters, and clinical data. This approach aids in building comprehensive management models, thereby improving the efficiency of DR diagnosis and treatment. The multimodal strategy not only enhances our understanding of DR’s pathophysiology but also offers new tools for early diagnosis and personalized treatment (13).

For instance, a research team developed the DeepDR-LLM system. This integrated intelligent system, which combines large language models with deep learning techniques, provides auxiliary diagnostic results for DR and personalized comprehensive diabetes management advice. Validated across multicenter cohorts in several countries, the system has proven effective in enhancing DR screening and primary diabetes management (65). Multimodal techniques also encompass the use of deep transfer learning for the automatic detection of DR images. By analyzing fundus images with pre-trained models, these techniques assist ophthalmologists in making diagnostic decisions. This method can detect DR in retinal fundus images automatically, without specifying lesion features, achieving high-precision predictions and reliable detection (66).

Advancements in technology have increased the efficiency of DR diagnosis and strengthened overall diabetes management, while also enabling multimodal imaging models to investigate links between retinal structure and neurodegenerative diseases (67). Concurrently, the FPRM dataset plays a critical role in developing high-precision, multimodal models by integrating ophthalmic and psychological data, thus enhancing machine learning’s understanding of the relationship between eye-health and psychological health (68).

The field of DR imaging research is progressively moving away from traditional diagnostic methods toward advanced computer vision and AI technologies. As AI continues to evolve, this shift is anticipated to intensify, potentially revolutionizing the conduct of multimodal DR imaging research and broadening its application to a wider spectrum of ophthalmic diseases.

### 4.4 Limitations

#### 4.4.1 Limitations of DR imaging research

There are still many insufficiencies in current DR imaging research. For instance, different research teams are limited by sample size when developing models; there are currently no comprehensive standards for diagnosing and grading different image information; existing intelligent auxiliary diagnosis and treatment systems still cannot independently complete the diagnosis and treatment tasks in ophthalmology. Technically, data privacy and security issues have become major challenges, and a more robust data encryption and secure transmission mechanism is needed. There are still many ethical restrictions in practical applications (69). Currently developed AI models for DR imaging are mainly based on single-image auxiliary diagnosis, and there is a lack of development of AI models for handling other modalities of ophthalmic data; in current ophthalmic AI research, the focus is mainly on fundus diseases, with relatively less research on other eye diseases.

It can be observed that the high-impact articles included mostly focus on the development of AI models related to DR imaging, and their numerous citations symbolize the high impact of these publications. The high volume of citations not only indicates that the research findings of the articles are widely disseminated and discussed but also may be because these studies play a crucial role at key points in the development of the field. At the same time, these high-impact articles also reflect the shortcomings in the development of the field. In subsequent research, the following shortcomings should be overcome as much as possible. After analyzing high-impact articles, we found that due to the complexity of DR image features, researchers find it difficult to accurately control the subtle details; intelligent algorithm systems can only show the level of the lesion and do not provide a clear definition of the disease from the fundus image (9); the multi-level analysis of intelligent algorithm systems is not unified with the clinical signs of the disease, and the actual promotion and application effects are greatly influenced by the acceptance of clinical ophthalmologists (70); the inconsistency of imaging equipment, individual differences between the examined, and the different degrees of their cooperation during the examination process lead to significant differences in the quality of images collected by different regions and medical institutions, which some extent affects the inclusion and feature annotation of the dataset in the research (71); some algorithms have insufficient generalization ability, specifically showing a downward trend in performance results when trained and validated in different databases (72); due to the lack of popularization and promotion of DR-related knowledge, the actual screening data collected in the work has limitations in representativeness and comprehensiveness, and cannot fully reflect the true situation of the disease; some DR imaging research may be limited due to a short follow-up time, making it difficult to fully assess the dynamic changes of the disease process.

#### 4.4.2 Limitations of bibliometric analysis

As a bibliometric study, compared with other ophthalmic bibliometric studies (6), this study standardized the search terms and focused the research direction on the field of DR imaging. At the same time, it included a broader time span of research in this field, providing a certain connection and supplement to the current bibliometric research in the field of ophthalmic imaging. At the same time, this study also has certain drawbacks and insufficiencies. In this study, we only searched and included English-language papers indexed in SCI and SCIE in the “WoSCC.” Therefore, we found that this study may miss some classic literature related to AI ophthalmology. Since the literature materials only selected literature indexed by SCI and SCIE and published in English, it may miss some original DR clinical application-related imaging research that was not included in the search scope. Bibliometric analysis focuses on the quantitative analysis of literature (such as citation frequency and publication quantity) and cannot directly measure the quality and impact of the research. The H-index, affected by publication timing, might not reflect a study’s long-term significance due to its failure to consider citation delays and differing citation trends among various disciplines (73). The latest research published by AI in ophthalmology requires a certain amount of time to be cited, and “citation lag” may make it difficult to see the influence of high-quality research in the early stage (74).

## 5 Conclusion

The United States is leading in research in this field. The University of London, University College London, and the National University of Singapore are the leading institutions in this field, with Invest. Ophthalmol. Vis. Sci. and Retin.-J. Retin. Vitr. Dis. being the journals with the highest publication volume. Tien Yin Wong and Klein, Ronald are the researchers with the highest publication volume and influence in this field. The hotspot most concerned by researchers is “deep learning,” and the keywords that have surged in the past 3 years are “deep learning,” “convolutional neural networks,” “artificial intelligence,” and “transfer learning.” In recent years, AI-related research represented by DL has become the latest hotspot. The field of DR imaging research is gradually shifting from traditional diagnostic methods to utilizing advanced computer vision and AI technologies to improve the accuracy and efficiency of diagnosis. The application of these technologies not only helps to better understand the pathophysiological mechanisms of DR but may also provide new tools for early diagnosis and personalized treatment. With the development of AI accompanied by imaging technology, future AI-involved multimodal DR imaging research may become the mainstream in this field and will involve a greater variety of eye diseases.

AI has achieved fruitful results in clinical applications in ophthalmology. The application of AI technology in clinical ophthalmology helps to improve the efficiency of diagnosis and treatment, reducing the repetitive work of doctors, especially in the screening and monitoring of eye diseases, which greatly alleviates the pressure on medical resources. Despite the many limitations and insufficiencies in the actual application process, the following points need to be addressed in the next related research: the generalization ability of the algorithm needs to be improved in the research to ensure its stable performance in different races, ages, and different eye diseases; the dataset needs to be expanded to ensure the application of the algorithm in a wide range of scenarios; the collaboration between AI technology and healthcare professionals needs to be improved to help medical workers better understand and use AI tools; the legal and ethical framework needs to be perfected, covering aspects such as data protection, patient rights priority, and algorithm transparency; promote the development of ophthalmic education to make medical workers more aware of AI so that it can be reasonably applied in clinical practice. With the continuous advancement of technology, the continuous upgrading of ophthalmic examination instruments, and the continuous improvement of datasets, the clinical application of AI in the field of DR imaging will continue to expand and bring more innovation and breakthroughs in the future.

## Data Availability

The original contributions presented in the study are included in the article/supplementary material, further inquiries can be directed to the corresponding authors.
